# Subclinical focal Cholangitis mimicking liver metastasis in asymptomatic patients with history of pancreatic Ductal Adenocarcinoma and Biliary tree intervention

**DOI:** 10.1186/s40644-017-0124-6

**Published:** 2017-07-14

**Authors:** Natally Horvat, Edmund M. Godfrey, Timothy J. Sadler, Jaclyn F. Hechtman, Laura H. Tang, Carlie S. Sigel, Serena Monti, Lorenzo Mannelli

**Affiliations:** 1 Department of Radiology, Memorial Sloan Kettering Cancer Center, 300 East 66th Street, New York, NY 10021 USA; 2 Department of Radiology, Hospital Sírio-Libanês, Adma Jafet 91, São Paulo, SP 01308050 Brazil; 3 Department of Radiology, Universidade de São Paulo, Dr. Enéas de Carvalho Aguiar, São Paulo, SP 05403900 Brazil; 4 Department of Radiology, Addenbrooke’s Hospital, Cambridge University NHS Foundation Trust, Hills Road, Cambridge, CB2 0QQ UK; 5 Department of Pathology, Memorial Sloan Kettering Cancer Center, 1275 York Avenue, New York, NY 10065 USA; 6 IRCCS SDN, Via E. Gianturco, 113, 80143 Naples, Italy

**Keywords:** Cholangitis, Pancreas cancer, Pancreatic ductal carcinoma, Biliary tract surgical procedures

## Abstract

**Background:**

Cholangitis is an inflammatory process of the biliary tract with a wide range of clinical manifestations and it is not always considered in the differential diagnosis in asymptomatic patients. To the best of our knowledge there is no previous report in the English literature of focal cholangitis manifesting exclusively as liver parenchymal changes mimicking liver metastasis in asymptomatic patients with pancreatic ductal adenocarcinoma (PDAC) and history of manipulation of the biliary tree. The purpose of this article is to present six cases of subclinical focal cholangitis mimicking liver metastasis in asymptomatic patients with history of PDAC and biliary tree intervention.

**Case presentation:**

There are six cases with new hepatic lesions detected on follow-up scans in asymptomatic patients with history of PDAC and manipulation of biliary tree. Overall seven lesions were detected, all of them were on the liver periphery, five were hypovascular and two were hypervascular. None of those patients had elevation of CA 19.9 compared with the previous exams. The three patients that had magnetic resonance imaging presented restriction on diffusion weighted imaging and high signal intensity on T2-weighted image. Two patients underwent liver biopsy, which showed only inflammatory changes. All patients were treated with antibiotics and underwent imaging follow-up, which demonstrated resolution of the lesions. None of the patients showed imaging or clinical signs of disease progression during this interval.

**Conclusion:**

Radiologists and oncologists need to be aware of the possibility of focal cholangitis causing hepatic lesions mimicking neoplasia in patients with history of biliary tree intervention, even in the absence of clinical symptoms.

## Background

Cholangitis is an inflammatory process of the biliary tract with a wide range of causes and heterogeneous clinical manifestations [1]. It can occur due to different infectious and noninfectious conditions, with its most common cause being biliary obstruction secondary to either tumour or bile duct stones. The sphincter of Oddi maintains sterility of the biliary ducts by preventing bacterial transposition from the duodenum [2]. Biliary tree intervention, such as biliary duct stenting, papillotomy, and biliodigestive anastomosis, is common in biliopancreatic disorders, and it is known to allow microorganisms to ascend into the biliary tree, increasing the risk of cholangitis [3].

Clinical manifestations of cholangitis may vary widely, from asymptomatic or oligosymptomatic to life-threatening scenarios. The classical Charcot’s triad (jaundice, fever and pain) and Reynolds pentad (when shock and lethargy are included) are not always present and make the clinical diagnosis challenging [4]. This can lead to delayed diagnosis or even misdiagnosis. Moreover, the imaging findings are nonspecific and can be due to bile duct or liver parenchyma abnormalities [5]. The most frequent bile duct changes are: (a) dilatation of biliary tree, which can be central, diffuse, or segmental, (b) thickening and enhancement of ductal walls, and (c) pneumobilia [5]. The liver parenchymal abnormalities are related to inflammatory process, causing dilatation of the peribiliary venous plexus, and to increased arterial flow [6, 7], which results in areas of increased signal intensity on T2-wighted images (WI) and/or abnormal enhancement on arterial phase, delayed phase, or both. Those areas can present wedge-shaped, peripheral or peribiliary distribution [5].

To the best of our knowledge there is no previous report in the English literature of focal cholangitis manifesting exclusively as liver lesions in asymptomatic patients with history of pancreatic ductal adenocarcinoma (PDAC). The purpose of this case series is to present six cases of subclinical focal cholangitis manifesting as new hepatic lesions in asymptomatic patients with history of PDAC and biliary tree intervention.

### Case presentation

**Fig. 1:**
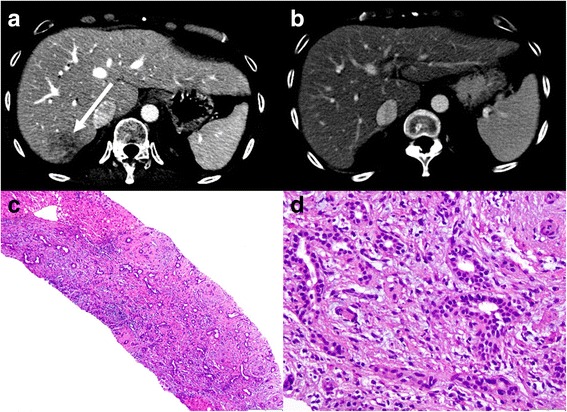
Case 1. **a** CECT demonstrates an ill-defined hypovascular area in the periphery of segment VII (*arrow*). Liver biopsy was performed with a diagnosis of inflammatory changes without malignancy. One month after the beginning of antibiotics, (**b**) CECT shows the resolution of the lesion. (**c**, **d**) Liver biopsy demonstrates prominent bile ductular proliferation, active cholangitis and portal oedema, with no malignant neoplasm

**Fig. 2:**
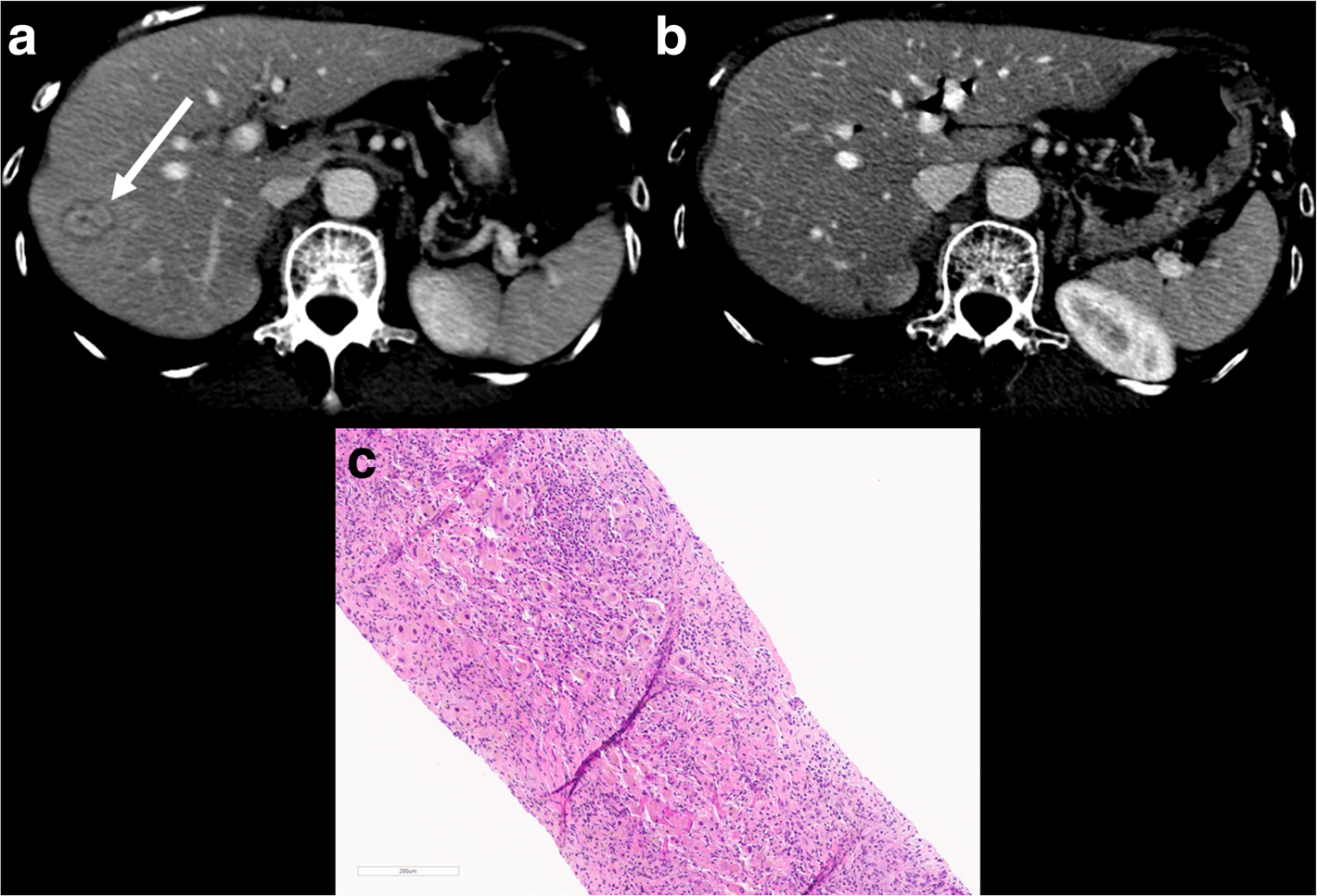
Case 2. **a** CECT shows a hypervascular nodule with target appearance in the periphery of segment VIII (*arrow*). The patient underwent liver biopsy with a diagnosis of inflammatory changes without malignant cells. **b** CECT 2 months after antibiotics the lesion was no longer identified. **c** Liver biopsy demonstrates a dense lymphoplasmacytic infiltrate of hepatic parenchyma with a paucity of bile ductules and no carcinoma is present

**Fig. 3:**
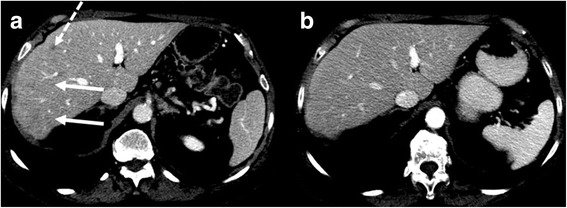
Case 3. **a** CECT demonstrates one hypovascular nodule in the periphery of segment IV (*dashed arrow*) and a hypovascular ill-defined area in the segments VII and VIII (*arrows*). Both lesions resolved on follow-up CECT (**b**)

**Fig. 4:**
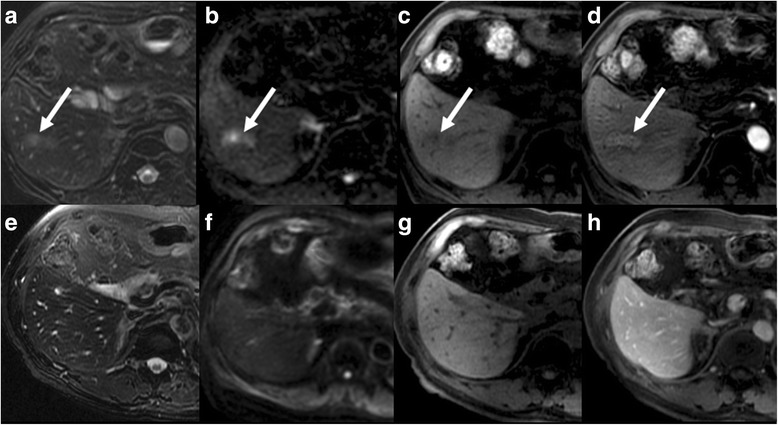
Case 4. MR images demonstrate an elongated lesion in the periphery of segment VI (*arrows*) with high SI on T2WI (**a**), restriction on DWI (**b**), low SI on T1WI (**c**), and post-contrast enhancement (**d**). The lesion resolved on follow-up MRI (**e-h**)

**Fig. 5:**
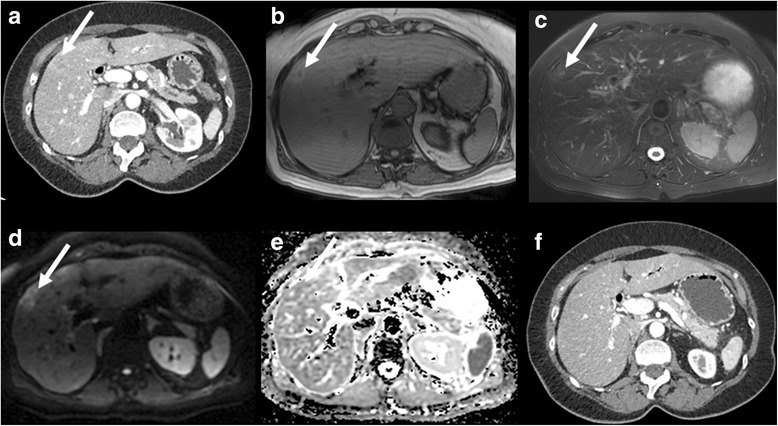
Case 5. CECT and MRI demonstrate a peripheral hypovascular lesion, surrounded by THAD (**a**), with high SI on T1WI (**b**) and T2WI (**c**), as well as restriction on DWI (**d**, **e**). The lesion resolved after 4 months of follow-up (**f**)

**Fig. 6:**
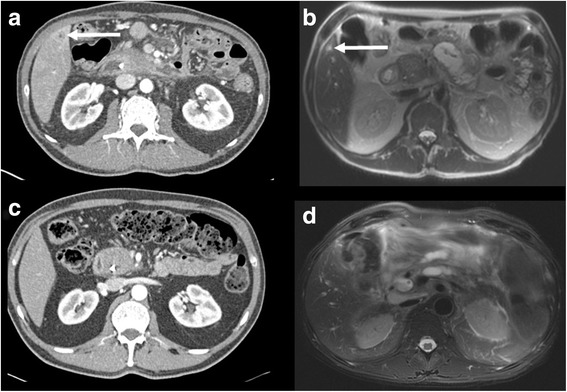
Case 6. CECT shows a peripheral hypovascular nodule with target appearance (*arrow*), surrounded by THAD (**a**). The nodule presented high SI on T2WI (**b**). After 3 months of follow-up the nodule was no longer demonstrated (**c**, **d**)

There are six cases with PDAC with new hepatic lesions on computed tomography (CT) and/or magnetic resonance imaging (MRI) detected on follow-up (Figs. 1, 2, 3, 4, 5 and 6): one patient after distal pancreatectomy, one patient after Whipple’s surgery, and four patients after starting chemotherapy for unresectable PDAC. None of the patients had previous liver metastasis or history of hepatic artery procedure. Clinical and imaging features of those patients are summarized in Table 1.

**Table 1:** Summary of focal cholangitis cases

Case	Sex	Age (years)	Symptoms	BTI	Interval between BTI and HL (days)	Laboratory abnormalities	CA 19.9 (U/mL) before / at diagnosis of HL	Imaging features	Outcome
1	F	57	None	BDA	558	None	17 / 19	CT (venous phase): ill-defined hypovascular lesion	Benign on LB and disappearance on FU
2	F	71	None	BDA	235	None	35 / 21	CT (biphasic): hypervascular nodule with target appearance in arterial and venous phases	Benign on LB and disappearance on FU
3	F	74	None	Papillotomy	99	None	34 / 38	CT (venous phase): hypovascular nodule (L1) and hypovascular ill-defined area (L2)	Disappearance on FU
4	M	69	None	BDA	479	None	52 / 18	MRI (triphasic): low SI on T1WI, high SI on T2WI, high SI on DWI, enhancement greater than liver background in all post contrast phases	Disappearance on FU
5	F	60	None	Stent	123	CA 19.9^a^	63 / 74	CT (biphasic): hypovascular lesion with peripheral THAD MRI (triphasic): high SI on T1WI, intermediate SI on T2WI, high SI on DWI, hypovascular in all post contrast phases	Disappearance on FU
6	M	59	None	Stent	165	CA 19.9^b^, AP^c^	2156 / 1032	CT (biphasic): hypovascular nodule with target appearance in arterial and venous phases and peripheral THAD MRI (triphasic): low SI on T1WI, high SI on T2WI, high SI on DWI, hypovascular in all post contrast phases	Disappearance on FU

*AP* alkaline phosphatase, *biphasic* arterial and venous phases, *BTI* biliary tree intervention, *CA* cancer antigen, *BDA* biliodigestive anastomosis, *DWI* diffusion weighted imaging, *FU* follow-up, *HL* hepatic lesion, *LB* liver biopsy, *L1* lesion 1, *L2* lesion 2, *SI* signal intensity, *THAD* transient hepatic attenuation differences, triphasic: arterial, venous and delayed phases

^a^CA 19.9: 74 U/mL (normal range: 0–37 U/mL)

^b^CA 19.9: 1032 U/mL (normal range: 0–37 U/mL)

^c^AP: 190 U/L (normal range: 45–129 U/L)

### Patient demographics and clinical data

Patients ranged in age between 59 and 74 years, with a mean age of 65 years and 4 (67%) were women. Two patients underwent partial pancreatectomy before the detection of the new hepatic lesions (case 2, 235 days of interval; case 4, 479 days of interval). All patients had history of biliary tree intervention, 3 biliodigestive anastomosis, 2 biliary duct stenting, and 1 papillotomy. None of the patients presented clinical symptoms at the time of the diagnosis of the hepatic lesions. Laboratory abnormalities were detected in 2 patients, both had elevated CA 19.9 (74 and 1032 U/mL, normal <37 U/mL) and 1 had elevated alkaline phosphatase (190 U/L, for a normal range of 45–129 U/L). However, these abnormalities did not present a significant increase in relation to previous exams.

### Imaging features

Three patients had only contrast-enhanced CT (CECT), one had only MRI and two had both CT and MRI. Overall, seven lesions were identified, five patients presented solitary lesions and one patient had two separate lesions. All lesions presented peripheral distribution, five were hypovascular and two were hypervascular on post-contrast phases. Five lesions were well-defined whereas two were ill-defined. On MRI, all three lesions presented restriction on diffusion weighted imaging (DWI) and high signal intensity (SI) on T2WI, and two lesions showed low SI on T1WI. Two nodules demonstrated target appearance on post-contrast images and two lesions presented peripheral transient hepatic attenuation differences. None of the patients had bile ducts changes such as dilatation or thickening.

### Outcome

Two patients underwent liver biopsy for further assessment of the new hepatic lesions, which showed inflammatory changes without malignant cells. All patients were treated with antibiotics and underwent imaging follow-up, which demonstrated resolution of the lesions. The mean time interval between the first scan that demonstrated the new hepatic lesions and the follow-up which no longer demonstrated them was 69 days (range, 39–112). None of the patients showed imaging or clinical signs of disease progression during this interval.

## Discussion and Conclusions

Infectious cholangitis is a potentially life-threatening condition, usually caused by an obstruction of the biliary tree [8]. Biliary tree intervention is also a relevant risk factor for cholangitis, by allowing microorganisms to ascend from the bowel to the biliary ducts [3]. The main imaging findings of cholangitis are related to bile duct abnormalities, such as dilatation, wall thickening and abnormal enhancement; pneumobilia may also be observed mainly after biliary tree intervention [5]. Nevertheless, liver parenchymal changes can also be demonstrated on imaging, including areas of increased signal intensity on T2WI and/or abnormal enhancement on arterial phase, delayed phase, or both. The treatment of cholangitis is based on antibiotic therapy and biliary drainage with decompression, if biliary obstruction is present [9, 10].

The fact that none of the patients of this case series showed clinical or laboratory signs of infection highlights that cholangitis can be asymptomatic, and imaging features may precede the clinical manifestation [4, 5], mainly in elderly patients [11]. None of the six patients had a significant increase in CA 19.9 levels at the time of diagnosis of the new hepatic lesions. Although, CA 19.9 was elevated in two patients, this raised the suspicion of progression or relapse of PDAC; however, and CA 19.9 increase can also be due to non-oncological causes, such as cholangitis and jaundice [12]. Furthermore, in these two patients that CA 19.9 was elevated, it was lower compared to prior measurement (one patient – case 6 had a significant reduction in compared to the previous exam), which makes the hypothesis of progression of neoplastic disease less likely.

The diagnosis of cholangitis can be challenging given the variability of clinical presentation and imaging findings, which are non-specific [4]. The challenge becomes even greater in clinical scenarios similar to those presented in this case series, in which patients with PDAC presented liver lesions without clinical symptoms or biliary tree abnormalities. The imaging features in our case series of focal cholangitis varied considerably, ranging from a hepatic hypervascular focus to an ill-defined hypovascular area, demonstrating that imaging findings alone cannot establish the final diagnosis. Even using functional imaging technique, such as DWI and PET, there will still be an overlapping between inflammatory and neoplastic changes [13–15]. A misdiagnosis of disease progression based solely on new hepatic lesions detected on CT or MRI can result in substantial changes in patients’ treatment, such as change in chemotherapy regimen and changes in eligibility criteria for clinical trials. For that reason, we speculate that in patients with PDAC and history of manipulation of the biliary tree, with no other unequivocal signs of disease progression and no significant rising of CA 19.9, it is reasonable to consider a differential diagnosis of cholangitis before deeming the hepatic lesion as metastasis. In cases where biopsy is not feasible, a therapeutic test and early imaging follow-up should be considered.

To the best of our knowledge there is no previous report in the English literature of focal cholangitis manifesting exclusively as liver parenchymal changes mimicking liver metastasis in patients with PDAC and history of manipulation of the biliary tree. Despite some limitations of this case series, such as the small number of patients and absence of histological confirmation in all cases, this manuscript could help radiologists when reporting similar cases and it could motivate further studies to better assess this entity. In this small group of patients, the diagnostic suspicion of focal cholangitis had a significant impact on clinical management.

Focal cholangitis may occur in asymptomatic patients with history of biliary tree intervention and can mimic hepatic focal lesions, such as metastasis. Radiologists and oncologists need to be aware of the possibility of focal liver parenchymal abnormalities caused by cholangitis, especially in patients with history of biliary tree intervention, because it is not always considered in the differential diagnosis by referring physicians given the lack of symptoms. Correlation with CA 19.9 and clinical status of the patient can help to achieve the correct diagnosis.

## Abbreviations

AP: Alkaline phosphatase
BTI: Biliary tree intervention
CA: Cancer antigen
CECT: Contrast-enhanced CT
CT: Computed tomography
BDA: Biliodigestive anastomosis
DWI: Diffusion weighted imaging
FU: Follow-up
HL: Hepatic lesion
L1: Lesion 1
L2: Lesion 2
LB: Liver biopsy
MRI: Magnetic resonance imaging
PDAC: Pancreatic ductal adenocarcinoma
PET: Positron emission tomography
SI: Signal intensity
THAD: Transient hepatic attenuation differences
WI: Weighted images
